# Heat Shock Proteins as Immunomodulants

**DOI:** 10.3390/molecules23112846

**Published:** 2018-11-01

**Authors:** Tawanda Zininga, Lebogang Ramatsui, Addmore Shonhai

**Affiliations:** Department of Biochemistry, University of Venda, P. Bag X5050, Thohoyandou 0950, South Africa; tzininga@gmail.com (T.Z.); lebogangramatsui@gmail.com (L.R.)

**Keywords:** heat shock protein, immune modulation, Hsp70, Hsp90, Hsp60

## Abstract

Heat shock proteins (Hsps) are conserved molecules whose main role is to facilitate folding of other proteins. Most Hsps are generally stress-inducible as they play a particularly important cytoprotective role in cells exposed to stressful conditions. Initially, Hsps were generally thought to occur intracellulary. However, recent work has shown that some Hsps are secreted to the cell exterior particularly in response to stress. For this reason, they are generally regarded as danger signaling biomarkers. In this way, they prompt the immune system to react to prevailing adverse cellular conditions. For example, their enhanced secretion by cancer cells facilitate targeting of these cells by natural killer cells. Notably, Hsps are implicated in both pro-inflammatory and anti-inflammatory responses. Their effects on immune cells depends on a number of aspects such as concentration of the respective Hsp species. In addition, various Hsp species exert unique effects on immune cells. Because of their conservation, Hsps are implicated in auto-immune diseases. Here we discuss the various metabolic pathways in which various Hsps manifest immune modulation. In addition, we discuss possible experimental variations that may account for contradictory reports on the immunomodulatory function of some Hsps.

## 1. Introduction

Heat shock proteins (Hsp) constitute part of the molecular chaperone (protein folding) system of the cell. Hsps are ubiquitous and occur in all celled organisms and they exhibit high level of conservation across species. Some but not all Hsps are stress inducible. Hsps are involved in several processes, amongst them, protein folding, protein trafficking, and protein complex assembly/disassembly [1]. Thus, Hsps play a central role in proteostasis. While some Hsps facilitate the folding of newly formed proteins, others are particularly induced during physiological stress in order to manage the extra burden of stress induced protein misfolding. In addition, Hsps are also involved in determining the fate of misfolded proteins by either refolding them or channeling them to the ubiquitin proteolysis pathway for degradation [2].

In general, most Hsps are expressed intracellularly. However, some Hsps are extruded to the cell surface particularly in response to physiological stress [3,4]. In addition, some Hsps of parasitic origin and antibodies that recognize them have been detected in the host circulatory system [4,5,6]. Thus, the Hsps of host and parasitic origin that end up in the host circulatory system ultimately interact with immune cells to modulate the function of the latter. Given their high conservation, Hsps are implicated not only in host immune modulation but are thought to play a part in the development of autoimmune conditions [7]. In addition, some Hsps are implicated in antigen processing and presentation [8]. Thus, Hsps are considered to be moonlighting molecules in light of their secondary roles outside their primary functions as molecular chaperones. In light of their growing attention as immune modulants, Hsps are implicated in various pathologies. This review seeks to highlight recent work on the role of Hsps in immune modulation. In addition, we discuss the various mechanisms by which they undertake this role. Furthermore, the role of these proteins in immune modulation has been shrouded in controversy on account of the divergent data reported for some Hsps with respect to their role in immune modulation. The contrasting views have largely emanated due to the use of recombinant Hsps preparations produced in *E. coli* which tend to be inherently tainted with lipopolisaccharides (LPS) which confound the downstream immune modulation studies. As such, in part this review seeks to reconcile findings from the various contradictory reports on the immunomodulatory role of some Hsps that could be due to technicalities associated with varying experimental designs.

### 1.1. Major Heat Shock Protein Families and Some of Their Roles in Immunomodulation

The classification of Hsps is mainly based on their molecular sizes [9]. They generally fall within seven major families: Hsp110, Hsp100, Hsp90, Hsp70, Hsp60, Hsp40 and small Hsps (approximately 15–30 kDa). Heat shock cognate (HSc) is a term that is used to describe the constitutively expressed forms of Hsps. On the other hand, most Hsps are induced in response to stress which make them important disease biomarkers [10,11,12]. Table 1, below provides a summary of the various immune modulatory pathways and pathological conditions in which some Hsps are implicated.

### 1.2. Heat Shock Proteins as ‘Chaperokines’

The term ‘chaperokine’ has been developed to describe molecular chaperones that play a role in signal transduction processes and immune modulation in general [20,21]. The role of Hsps such as Hsp60, Hsp70, Hsp90, and an ER based Hsp70 homologue, glucose regulated protein 96 (gp96) in the production of pro-inflammatory cytokines has been reported [22]. Some of the cytokines that are produced in response to the presence of Hsps include tumor necrosis factor (TNF-α), interleukin (IL)-1, IL-6, and IL-12 and anti-inflammatory cytokines such as IL-10 [23,24]. Furthermore, some Hsps induce the release of nitric oxide (NO), C-C chemokines by immune cells [25]. Hsps are also thought to modulate maturation of dendritic cells [26,27].

### 1.3. Hsp60

**Figure 1:**
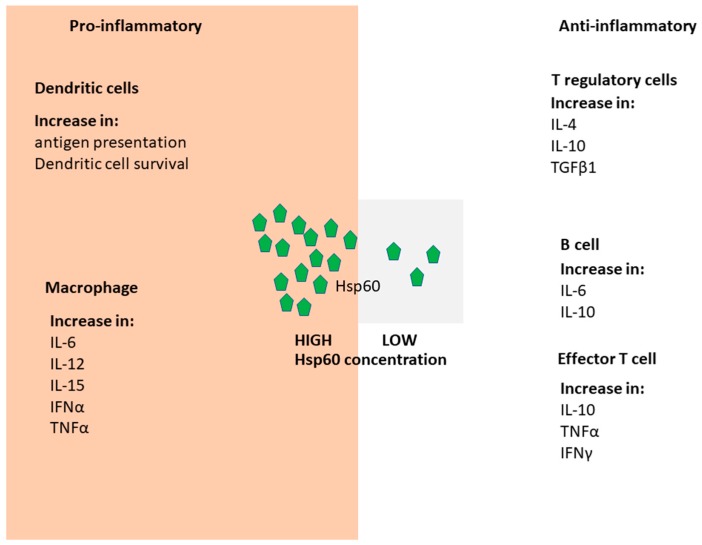
Hsp60 immunomodulatory pathways. Hsp60 is recognized by receptors of both the innate and adaptive immune systems. The receptors bind Hsp60 to initiate signal transduction giving rise to the production of effector cells and cytokines. Depending on the levels of Hsp60 present, pro-inflammatory or anti-inflammatory responses are initiated. The pro-inflammatory events result in the survival of dendritic cells and enhanced maturation of macrophages. On the other hand, the anti-inflammatory events result in suppressed activity of T regulatory cells and reduced migration of effector T cells.

The primary role of Hsp60 is to actively fold unfolded protein substrates localized to the mitochondria, while its cytosol isoform is termed, Tailless Complex Peptide (TCP/TRiC) [28]. Although Hsp60 is generally an intracellular molecule, its release into extracellular space has been reported to occur through physiological secretion as well as on account of cell necrosis [29,30]. Hsp60 generally function as a tetradecamer of back to back, seven membered rings of 60 kDa subunits [31,32]. The 10 kDa Hsp10 monomer forms heptameric complex providing a lid that closes the Hsp60 oligomer opening [31,32]. Notably, the *Mycobacterium tuberculosis* Hsp60/Hsp10 complex does not exhibit the seven-fold symmetry [33]. Instead, it forms a cage into which the unfolded peptides are trapped and allowed to fold. Hsp60 circulating in blood serves as a signal for macrophages and dendritic cells [29,34]. It is thought that Hsp60 modulates the innate immune system via activation of TLR4 and TLR2 [29]). Hsp60 serves dual and contrasting functions. It is thought to be pro-inflammatory via its effects on monocytes, B cells and effector T cells, while it exhibits anti-inflammatory functions on B cells, T regulatory cells (Tregs) cells and antiergotypic T cells [29]. In this respect, its effect on the immune system is deemed to be a function of concentration (Figure 1).

Hsp10 has also been found to be upregulated and is independently implicated in immune modulation [35]. Hsp60 and Hsp10 have been described as early pregnancy factors [36]. In this regard, the enhanced presence of Hsp60 in sera of pregnant women is linked to immunosuppression which enhance tolerance of the fetus [36,37]. In addition, because of its role in immune suppression, Hsp60 is known to facilitate skin grafting [35]. Furthermore, Hsp60 was implicated in the development of autoimmune encephalomyelitis in rats [29]. However, the mechanism by which it plays this role is not known. Intervention studies targeting Hsp60 function in various human inflammatory diseases have been reported [29,38].

*Mycobacterium tuberculosis* Hsp60 has been shown to elicit a proinflammatory response in cultured human vascular endothelial cells by inducing synthesis of leukocyte adhesion receptors [39]. Extracellular Hsp60 was reported to promote cell proliferation and regeneration of hair cells and caudal fins of zebrafish [40]. This function is thought to be linked to its capacity to promote the M2 phase of macrophages.

In addition, Hsp60s from *Helicobacter pylori* stimulate a pro-inflammatory immune response through their interaction with TLR4 or TLR2 independent of LPS or PAMS [41]. It is interesting to note that although Hsp60 is highly conserved, the effect of various Hsp60 isoforms on the immune cells is distinct. For example, despite *M. tuberculosis* Hsp60.1 (MtbHsp60.1) and its homologue, Hsp60.2 (MtbHsp60.2) sharing 76% sequence identity, MtbHsp60-1 activates monocytes much more effectively than MtbHsp60-2 [42]. In addition, MtbHsp60-1 also inhibits allergic asthma in mice, whilst MtbHsp60.2 and bacterial Hsp60s have no effect on allergy [43]. While, MtbHsp60.1 was reported to stimulate epithelioid cells and Langhans giant cells, MtbHsp60.2 served as an adhesin to facilitate binding of the bacterium to macrophages as part of the immune cell invasion process [44]. 

Several auto immune diseases have been associated with significant presence of antibodies that recognize Hsps [29]. For example, antibodies that reacted with Hsp60 from various infectious microorganisms were reported in patients suffering from spondylarthrosis which suggests a role for Hsp60 from infectious agents in autoimmune diseases [45,46,47,48,49]. It should be noted however, that the apparent detection of the various Hsp60 homologues from various species could have been due to cross-reactivity of the antibodies as they appear to bind to a common conserved epitope [50,51].

Some Hsp60s of eukaryotic origin induce both anti-inflammatory and pro-inflammatory events. Elevated expression of Hsp60 has been reported to correlate with the production of IL-10 and IL-4 which suppress the severity of rheumatoid arthritis [52]. In addition, Hsp60 activates CD30+ T-cells and CD4+CD25+ regulatory T-cells via cell exposed TLR2 to secrete IL-4 and IL-10 [53]. These events are responsible for the suppression of juvenile idiopathic arthritis [54], and several autoimmune diseases such as diabetes and rheumatoid arthritis [3,53]. Human Hsp60 is implicated in pro-inflammation through its capability to stimulate macrophages to secrete IL-6; TNF-α, IL-12 and IL-15 [55]. It is interesting to note that Hsp60 of host origin is thought to serve as a receptor for some pathogens. For example, *Listeria monocytogenes* adhesin alcohol acetaldehyde dehydrogenase binds to Hsp60 located on enteric epithelial cells [56]. Taken together, Hsp60 a potent immunomodulatory molecule as it is involved in several pathways determining the type of immune response.

### 1.4. Hsp90

The Hsp90 chaperones are predominantly intracellular molecules that are involved in facilitating maturation of several receptors and kinases. Hsp90 is one of the most ubiquitous chaperones accounting for 1–2% of the cell proteome [57]. Hsp90 occurs in 4 forms in eukaryotes. Of these, two are cytosolic isoforms (one is inducible, and the other is constitutive). The other two are localized in the ER and mitochondrion. The presence of cytosolic Hsp90 in the extracellular matrix originates through either secretion via exosomes [58,59] or exposure of the cell membrane bound form of the protein to the extracellular matrix [60]. The secretion of the inducible, cytosolic isoform of Hsp90 (Hsp90-α) into the extracellular matrix is associated with cell stress and phosphorylation of its Thr-90 residue [61]. Extracellular Hsp90 is thought to facilitate the folding and hence the activity of receptors on immune cells such as natural killer cells and T lymphocytes [62]. Topical application of Hsp90 promotes wound healing [63], and this is thought to occur through Hsp90-mediated phagocytosis of the wound debris [64]. Surface exposed Hsp90 serves as a signal for danger/damage associated molecular patterns (DAMPs) which activates the innate immune response, and ultimately the adaptive immune system [65]. Thus, Hsp90 serves as a signal to alert immune cells to the presence of cancer cells leading to their death [62,64].

**Figure 2:**
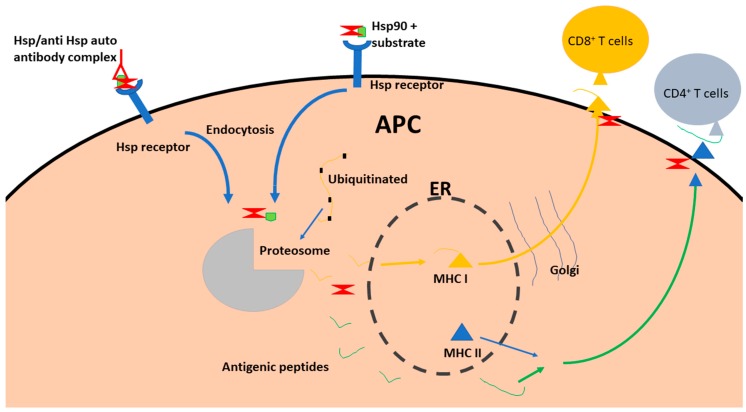
Schematic of the role of Hsp90 in antigen presentation. Extracellular Hsp90 binds to peptide substrate (in autoimmunity, Hsp90 comes already bound to antibody) and the Hsp90 is recruited by Hsp receptors on APC. Then the Hsp90 and bound substrate are internalized through vesicles to be processed by the proteasome. After processing these antigenic peptides are loaded onto MHC II released from the ER. The MHC II and peptides are transported to the cell surface for presentation to CD4+ T cells. Intracellular Hsps are involved in endocytosis and processing of the antigenic peptides (such as delivery to the proteasome subunits and MHCI). The generated peptides are processed through the ER and subsequently presented to CD8+ T cells.

Both intracellular and extracellular Hsp90 are involved in antigen presentation [62]. The intracellular form of Hsp90 binds to antigenic proteins to facilitate their processing to antigenic peptides. The generated antigenic peptides are eventually presented to MHC-I/II by Hsp90 [62]. On the other hand, extracellular Hsp90 binds to substrate peptide antigens and interact with cell surface receptors to facilitate uptake of the Hsp90-antigen complex by endocytosis [66]. In this way, Hsp90 acts as an adjuvant. Following the internalization of the antigens, intracellular Hsp90 facilitates passage of the peptides to the proteasome for degradation [66]. After cleavage into small peptides 7–14 residues long the antigenic peptides are then translocated to the ER. Some of the antigenic peptides are secreted through the ER-Golgi pathway [66]. Thus, Hsp90 facilitates cross-presentation of antigens to immune cells [67]. Overall, the role of Hsp90 in immune cell modulation is dependent on its various functions such as chaperoning of peptides, complexing with them and to facilitate their delivery to immune cells (Figure 2).

### 1.5. Hsp40

Heat shock protein 40 (Hsp40) family members (called DnaJ in prokaryotes) possess a highly conserved J-domain comprising of approximately 70 amino acids [68]. The J domain houses an HPD motif that facilitates interaction of the co-chaperone with Hsp70 chaperones. Generally, Hsp40s are grouped into four types (I-IV) mainly based on their structure-function characteristics [69]. Type I and type II Hsp40s have the capability to bind substrates through their CXXCXGXG (CRR) motif and as well as the C-terminal region [69], but they cannot refold the substrates. Hence, they facilitate delivery of misfolded protein substrates to Hsp70 for refolding. However, both type I and II Hsp40s are capable of independently suppressing protein aggregation [69]. Type I and II Hsp40s associate with Hsp70s to stimulate the latter’s ATPase activity [70]. Thus, interaction of Hsp40 with Hsp70 involves two aspects; substrate recruitment with concomitant stimulation of the ATPase activity of Hsp70 [71]. The interaction between Hsp70 and Hsp40 is mediated through the J domain of the Hsp40 [71]. The number of Hsp40 isotypes present in organisms generally increases with cell complexity. Thus, eukaryotic cells generally express an expanded arsenal of Hsp40s compared to prokaryotes. For example, *E. coli* possesses 4 Hsp40s while human genome hosts more than 50 genes encoding for Hsp40-like proteins. Interestingly, the malaria parasite genome harbors 51 Hsp40 genes [69,72]. It has been reported that at least 19 of *P. falciparum* Hsp40s could be exported to the malaria-parasite infected red blood cell where they are implicated in host cell remodeling leading to malaria pathology [69,72]. Some of the proteins originating from the malaria parasite that are trafficked to the infected host red blood cell are thought to associate with some host cells forming knobs that are exposed on the red blood cell membrane surface. The knobs are implicated in malaria pathogenicity as they facilitate adhesion of the infected red blood cells to endothelial cells. Due to their cell surface exposure, these knobs are targeted by the immune system. On the other hand, by promoting adhesion of the parasite-infected red blood cells to endothelial membranes, the knobs thus facilitate evasion of the immune system by the parasite-infected red blood cells [73].

The direct role of Hsp40s in immune modulation has not been well studied. A *Streptococcus pneumoniae* Hsp40 has been shown to stimulate macrophages to secrete IL-6 through activation of PI3K and JNK signaling pathway towards a pro-inflammatory response [13]. In their attempt to validate that the immuno-stimulation by their recombinant *S. pneumoniae* Hsp40 preparation was not due to LPS contamination, Cui et al. [13], passed the protein through a column enriched with polymyxin B (PMB). The eluted protein retained its capability to stimulate IL-6 production in cells cultured in vitro suggesting that Hsp40 may modulate immune cells. Another independent study reported elevated presence of antibodies that recognize both *E. coli* Hsp40 (DnaJ) and human Hsp40 (Hdj1) in patients suffering from rheumatoid arthritis [74]. This led the group to conclude that both DnaJ and Hdj1 significantly inhibited the replication of CD4+ and CD8+ T cells. Furthermore, the two proteins are implicated in the induced secretion of the main anti-inflammatory cytokine, IL-10 by PBMCs of patients suffering from rheumatoid arthritis, suggesting a role of these two Hsp40s as natural suppressors of inflammation.

### 1.6. Hsp70

**Figure 3:**
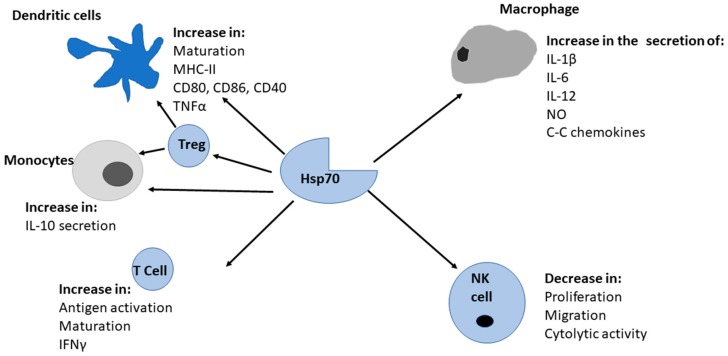
Hsp70s stimulation of immunity. Hsp70 stimulates both the innate and adaptive immune systems. The recognition of Hsp70 by immune cells causes initiation of signal transduction which results in the subsequent release of cytokines.

Hsp70 (called DnaK in prokaryotes) is a ubiquitous family of molecular chaperones that plays a central role in proteostasis [75]. While some Hsp70s are constitutively (HSc70s) expressed to serve as house-keepers, others are stress inducible. Hsp70 is generally composed of an N-terminal 44 kDa nucleotide binding domain (NBD) which exhibits ATPase activity, substrate binding domain (SBD) and a C-terminal lid. In canonical (DnaK-like) Hsp70s, the NBD and the SBD are connected by a highly conserved 7-residue linker region [76]. Hsp70s are divided into two sub-families: DnaK-like (canonical Hsp70s) and Hsp110 [77]). The canonical Hsp70s are capable of refolding misfolded protein and suppressing protein aggregation [78]. On the other hand, Hsp110 members are specialized proteins that are structurally distinct as they possess an extended lid segment compared to canonical Hsp70s. Despite most of them not possessing leader sequences for export, most Hsp70s are released from the cell interior to the extracellular space [79]. The release of Hsp70 to the extracellular matrix is triggered by cell stress and the accumulation of the chaperone in extracellular space serves as a danger signal to the immune system [79]. Extracellular membranes containing Hsp70 were found to induce TNF-α production by macrophages by up to 260-fold [79]). In addition, Hsp70 facilitates delivery of antigen to T cells [80,81]. The reported specific effects of Hsp70 on various immune cells is summarized in Figure 3.

It has been established that extracellular Hsp70 is released from cells via several processes amongst them, active secretion, release from dead cells and membrane binding [82]. The active secretion of Hsp70 is mediated by its initial association with lipid raft glycolipid globoyltriaosylceramide (Gb3) [83]. Hsp70 is then ultimately taken to extracellular space via its subsequent association with phosphatidylserine (PS) [84]. It is thought that following exposure to cellular stress, PS associates with Hsp70 to facilitate the latter’s translocation from the inner to the outer plasma membrane through the action of Ca2+ and ATP dependent phospholipid scramblase [85]. The purpose of translocating PS to the outer membrane is not fully known. However, externalization of PS acts as a ‘danger signal’ that alerts macrophages to respond [86]. The same mechanism for release of Hsp70 to the external cellular environment is thought to account for the deposition of Hsp70 to the exterior of cancer cells [83]. Extracellular Hsp70 is thought to facilitate pro-inflammatory response of immune cells by inducing cytokines [87]. In contrast, membrane-bound and extracellular Hsp70 is a potent modulator of innate immune system through its ability to bind to specific recognition sites located in the plasma membrane of activated natural killer cells [88].

**Figure 4:**
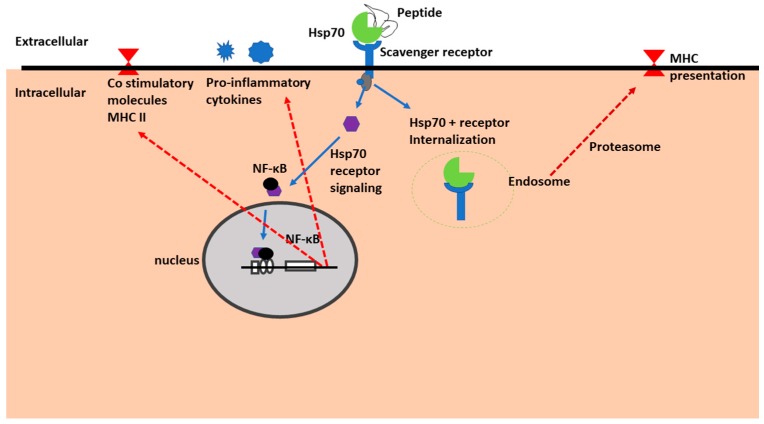
Immunomodulatory function of extracellular Hsp70. Hsp70 bound to peptide substrates is recognized by the scavenger Hsp70 receptor. This generates a signal that causes NF-κB to translocate into the nucleus. Once in the nucleus, NF-κB activates synthesis of pro-inflammatory cytokines and immune cell co-stimulation through MHC II. An alternative pathway for Hsp70-peptide-receptor complex internalization is through endocytosis, leading to subsequent processing of the peptide by proteasomes to generate antigenic peptides that are presented on MHC II.

Binding of Hsp70 to natural killer cells is thought to occur via the scavenger receptor family as well as via C-type lectins [89]. In addition, the same study confirmed that various scavenger receptor family members were able to facilitate cellular uptake of Hsp70-peptide complexes [89]. The scavenger receptors once bound to Hsp70 initiate signal transduction resulting in activation of transcription factor NF-κB to translocate to the nucleus where it initiates transcription and release of pro-inflammatory cytokines (Figure 4). The Hsp70 pro-inflammatory activity is linked to the entry of transcription factor NF-κB into the nucleus where it enhances transcription of genes resulting in the increased secretion of TNF-α, IFN-γ, IL-1β, I-L6 and IL-12 [90,91]. On the other hand, its anti-inflammatory function is linked to its capability to activate IL-10 production by restricting entry of NF-κB into the nucleus [15].

*Plasmodium falciparum*, the main agent of malaria expresses six Hsp70s, and antibodies that recognize two of these of these proteins: PfHsp70-1 and PfHsp70-x [92,93]; have been reported to be present in sera obtained from individual living in malaria endemic areas [6]. This suggests a possible role for these two proteins of parasitic origin in host immune modulation. However, a recent study could not confirm the immune modulatory activity of recombinant PfHsp70-1 protein on immune cells that were cultured in vitro [94]. It is possible that the production of recombinant PfHsp70-1 in *E. coli* which lacks post-translational modification capability of proteins may explain the observed apparent lack of immunomodulatory activity of PfHsp70-1.

### 1.7. Hsp70 as Receptors

The immune-regulatory functions of natural killer cells is to kill target cells via secretion of IFN-γ, TNF-α, release of granzyme B (GrB) and perforin [95]. It has been shown that membrane bound Hsp70 acts as docking sites for the scavenger receptors such as the activatory C-type lectin receptors of natural killer cells [16]. In contrast, to T cells, natural killer cells are thought to recognize membrane bound Hsp70 that is void of immunogenic peptides [82,96]. This pathway stimulates natural killer cells to release GrB. The activation of natural killer cells is thought to be facilitated by a TKD (TKDNNLLGRFELSG, aa 450–463) motif of Hsp70 [97]. The GrB released by the natural killer cells is glycosylated and this facilitates its recognition by membrane bound Hsp70 on cancer cells [98]. The membrane bound Hsp70 promotes GrB internalization by the surface Hsp70 positive tumor cells [99]. Thus, the surface exposure of Hsp70 by tumor cells enhances their sensitivity to cytolytic attack by natural killer cells.

It is thought that Hsp70 bound onto the infected red blood cell membrane is thought to interact with GrB, leading to its uptake and subsequent selective lysis of the malaria parasite-infected red blood cells [100]. *P. falciparum* infected red blood cells harbor Hsp70 of human origin as well as the exported parasite protein, PfHsp70-x [6,93]. Thus, it is plausible that both human Hsp70 and PfHsp70-x may interact with natural killer cells to stimulate GrB release and leading to selective lysis of parasite infected red blood cells [16]. Such a prospect raises the possibility of exploring GrB-mediated malaria therapy.

Several studies on the immune modulatory function of bacterial Hsp70s has been based on *M. tuberculosis* Hsp70 [101,102]. MtbHsp70 binds to CD40 receptors on human monocyte cells [103]. It is the C-terminal domain residues 407–426 that are deemed responsible for activating dendritic cells [103]. MtbHsp70 interacts with CCR5 receptor [104]. This CCR5 binding epitope is reported to block the uptake of HIV, making it a potential therapeutic target [105]. Interestingly, MtbHsp70 C-terminal residues (residues 457–496) inhibited differentiation of dendritic cells [103]. The contrasting immunomodulatory functions of various C-terminal fragments of MtbHsp70 are thought to be on account of their capability to unique modulate the activity of the p38 MAPK isoform [103].

### 1.8. Small Hsps

Small heat shock proteins are important in cytoprotection against stress. Apart from their role as molecular chaperones, it has been established that some disease conditions, amongst them cancer modulate their expression. It has been reported that small heat shock proteins such as Hsp27 are phosphorylated by the kinases, MAPK and Akt [106]. It was further proposed that Akt and Hsp27 associate and that this interaction is crucial for activation of neutrophils by Akt [107]. In addition, the phosphorylation of Hsp27 by Akt is important for dissociation of the two molecules [107]. Furthermore, the dissociation of the two molecules promoted apoptosis of neutrophils and polymorphonuclear leukocytes [107,108]. In addition, Hsp27 is thought to reduce cholesterol uptake by macrophages through its capability to bind scavenger receptor-A and thus it is implicated in suppressing vascular inflammation [109].

### 1.9. Contaminating Constituents that Confound Immunomodulation Studies

A number of studies reporting on the immune modulation function of Hsps are based on the use of recombinant Hsps produced in *E. coli*. The presence of LPS contaminants in the recombinant proteins used in such assays confounds the data as LPS is a potent immune cell modulant (Table 2). For example, it has been reported that people living in malaria endemic areas possess antibodies that recognize two malarial heat shock proteins, *P. falciparum* Hsp70-1 (PfHsp70-1) and PfHsp70-x suggesting that these two chaperones possess immunomodulatory properties [6,110]. On the other hand, a study by Pooe and colleagues [94], showed that recombinant PfHsp70-1 lacks immune modulatory activity when it was introduced to immune cells cultured in vitro. It has been proposed that post-translational modification of some proteins is essential for their immune modulator function [111]. Thus, the reported lack of immune modulatory capability demonstrated by recombinant PfHsp70-1 could be because it was not post-translationally modified. However, several studies have suggested that Hsp70 is an immunomodulatory agent [29,91]. Therefore, the role of various contaminants of recombinant heat shock proteins, amongst them LPS, flagellin and nucleotides in stimulating immune cells has been highlighted (Table 2).

To reduce the masking effect of LPS on immune stimulation of Hsp70, several studies have used polymyxin B (PMB) to bind LPS and thereby removing it from the purified recombinant proteins [12,13,119]. While, this may be effective for some proteins, we recently noted that passing recombinant Hsp70 through a column charged with ATP helps to further strip the protein of LPS contamination [94]. This suggests that PMB is inadequate to remove LPS that may be lodged within the core of the Hsp70 or possibly bound in the substrate binding cavity of Hsp70. While the use of PMB has been broadly used to strip recombinant proteins used in immune modulation of LPS, the effect of PMB itself on the function of Hsp70 is poorly understood. To this end, we recently conducted a study that demonstrated that PMB binds to Hsp70 to effect structural perturbations on the protein [120]. In fact, PMB, binds Hsp70 to inhibit the chaperone’s association with functional protein interactors [120]. These findings suggest that PMB may cause adverse structural changes to proteins which would further confound its application in removing LPS from recombinant protein preparations intended for down-stream immune modulatory studies. Since PMB interferes with association of Hsp70 with functional partners, it is possible that subjecting Hsp70 to PMB treatment may abrogate the association of the protein with immune cell receptors leading to false negative results. Since the pro-inflammatory activity of Hsp70s is confounded by LPS contamination, stripping recombinant Hsp70 of LPS resulted in the switch of its role from stimulation to immuno-suppression as reported based on a study that was conducted using a mouse model [121]. Altogether, contribution of contaminants that occur in preparations of recombinant Hsps used in immunomodulatory studies accounts in part for the controversy linked to the divergent reported findings with respect to the role of these proteins. Nonetheless, a growing body of data suggest that most of them exhibit capability to modulate the function of immune cells. There is urgent need to improve methods used to purify recombinant proteins for their subsequent application in immunomodulatory studies.

## 2. Conclusions

Heat shock proteins are implicated in a myriad of immune modulation functions. Interestingly, the effect of some heat shock proteins on the function of immune cells yielded contradictory outcomes. In some instances, this was attributed to unique experimental settings such as concentrations of the respective protein as the effect of some of these proteins depends on their levels. In some instances, the varied outcomes are due to contaminating species accompanying the respective heat shock proteins under study. In light of this, there is need to improve the quality of proteins preparation used in immunomodulation. Furthermore, it is interesting to note that nearly all major families of heat shock proteins function in networks. It remains to be investigated how their cooperation influence their role in the modulation of immunity. However, to date the studies that have been conducted have opened opportunities for the possible pursuit of these molecules in intervention against conditions such as cancer and autoimmune diseases. This is because immunization studies using animal models have demonstrated capability of some heat shock proteins to confer lasting protective effects against some chronic and infectious diseases. For example, mycobacterial Hsp60 and Hsp70 were shown to induce anti-inflammatory factors such as IL-10 expression by T cells [67,102]. It is thus not surprising that some heat shock proteins have entered the clinical trials pipe-line against conditions such as rheumatoid arthritis and autoimmune type I diabetes [122,123]. There is no question that the focus on heat shock proteins as immune modulators towards clinical interventions will continue to grow.

## Figures and Tables

**Table 1:** Role of heat shock proteins in immunomodulation and aligned pathologies.

Proteins	Associated Pathology	Immunomodulatory Function	Ref
sHsps	Cancer	Phosphorylation of Hsp27 by mitogen-activated protein 2 (MK2) is implicated in signal transduction. Hsp27 regulates Akt (protein kinase B) activation.	[13]
Hsp40	Pneumococcal infection	Stimulates Th1 and Th17 immune response against *Streptococcus pneumoniae* infection in mice. Activates BMDCs through recognition of TLR4 causing activation of MAPKs, NF-κB and PI3K-Akt pathways resulting in secretion of IFN-γ and IL-17A.	[14]
		Induces pro-inflammatory cytokine production in macrophages. Activates PI3K and JNK signal pathways resulting in secretion of IL6.	[15]
Hsp60	Type 1 diabetes mellitus	Induces both pro-inflammatory and anti-inflammatory cytokines. Binds multiple allelic variants of HLA-DR, this results in the release of IL-10, an anti-inflammatory cytokine, and IFN-γ.	[3]
	Type 2 diabetes	Interaction of Hsp60 with TLR2 and TLR4 results in release of pro-inflammatory cytokines (IL-1β, IL-6, IL-8, MCP-1 and TNF-α).	[16]
Hsp70	Chronic inflammatory diseases	Promotes the production of anti-inflammatory cytokines. Interact with DCs, MDSCs, and monocytes, by binding to their endocytic receptors resulting in the release of anti-inflammatory cytokine IL-10 and inevitable immunosuppression.	[17]
	Cancer	Acts as extracellular localized recognition site for NK cells. Interaction with NK cells through the TKD motif results in cytolytic attack mediated by NK cells.	[18]
Hsp90	Cancer	Hsp90 is implicated in T-cell mediated antitumor responses. Hsp90 inhibition up-regulates expression of interferon response genes, which promotes killing of melanoma cells by T cells.	[19]

Keywords: Bone marrow-derived dendritic cells (BMDCs), c-Jun N-terminal kinase (JNK); dendritic cells (DC); human major histocompatibility complex molecule (HLA); interferon-γ (IFN-γ); interleukin (IL); Mitogen-activated protein kinases (MAPK); Monocyte chemoattractant protein-1 (MCP-1); myeloid-derived suppressor cells (MDSC); natural killer cells (NK); Phosphatidylinositol 3-kinase (PI3K); T helper cells (Th); Toll-like receptor 2/4 (TLR2/4); Tumor necrosis factor-α (TNF-α).

**Table 2:** Role of LPS and flagellin based contaminants in studies investigating immunomodulatory function of recombinant heat shock proteins.

Heat Shock Protein Implicated in Immunomodulation E	Contaminants Implicated in Study	References
Hsp60 induced pro-inflammatory cytokine production by macrophages	LPS	[20,112]
Hsp70 induced pro-inflammatory cytokine production by macrophages	LPS	[94,113]
Hsp70 induced activation and maturation of dendritic cells	LPS	[114]
Hsp70 and anti-CD-3 co-stimulation of IL-2 production by Jurkat T cells	Flagellin	[115]
Hsp70 induced, CCR5 mediated calcium signaling by dendritic cells	Nucleotides (ATP and ADP)	[116]
Hsp90 induced pro-inflammatory cytokine production by macrophages	LPS	[117]
Gp96 induced activation of NF-κB and production of NO by macrophages	LPS	[118]
